# Effect of HongJing I in Treating Erectile Function and Regulating RhoA Pathway in a Rat Model of Bilateral Cavernous Nerve Injury

**DOI:** 10.1155/2019/1083737

**Published:** 2019-09-16

**Authors:** Miao-yong Ye, Fan Zhao, Ke Ma, Kang Zhou, Wen-Jie Huang, Yin-feng Ma, Jian-feng Zhao, Hui-ying Fu, Zeng-bao Xu, Bo-dong Lv

**Affiliations:** ^1^The Second Clinical Medical College, Zhejiang Chinese Medical University, Hangzhou 310053, China; ^2^Department of Urology and Andrology, Affiliated Hospital of Nantong University, Nantong 226001, China; ^3^Department of Urology and Andrology, The Second Affiliated Hospital of Zhejiang Chinese Medical University, Hangzhou 310053, China; ^4^Research Institute of Urology and Andrology, Zhejiang Chinese Medical University, Hangzhou, 310053, China; ^5^Department of Urology, Huzhou Hospital of Traditional Chinese Medicine, Huzhou 313000, China; ^6^Andrology Laboratory on Integration of Chinese and Western Medicine, Zhejiang Provincial Key Laboratory of Traditional Chinese Medicine, Hangzhou 310053, China

## Abstract

HongJing I (HJI), a traditional Chinese herbal formula, has been confirmed to be effective for the clinical treatment of erectile dysfunction (ED). However, the mechanism of action of HJI remains unclear. Here, we aimed to investigate the effect and underlying mechanisms of HJI against ED in a rat model of bilateral cavernous nerve injury (BCNI). Rats were divided into five groups: normal control (NC), BCNI-induced ED model (M), M + low-dose HJI (HL), M + medium-dose HJI (HM), and M + high-dose HJI (HH). All groups were treated with normal saline or the relevant drug for 28 consecutive days after inducing BCNI-ED. At the end of the treatment period, the intracavernous pressure (ICP) was recorded, and histological examination was conducted using Masson's trichrome staining. Immunofluorescence staining and western blotting were applied to detect the changes in fibrosis protein and Ras homolog A (RhoA), Rho-associated protein kinase 1 (ROCK1), and ROCK2 expression. We found that HJI effectively improved the ICP in the treatment groups. In addition, RhoA, ROCK1, and ROCK2 expression levels were increased upon BCNI-ED induction, and HJI successfully inhibited cavernosum fibrosis and the activation of RhoA/ROCK2 signaling. Overall, these results suggest that the effects of HJI in attenuating ED may be caused, at least in part, by the suppression of RhoA/ROCK2 signaling and alleviation of fibrosis. However, the precise mechanism surrounding this requires further investigation in future studies.

## 1. Introduction

Erectile dysfunction (ED) remains a common consequence of radical pelvic surgeries such as radical prostatectomy, despite the development of effective surgical techniques [1]. Cavernous nerve (CN) injury is a common result, which leads to neuropraxia and the damage and dysfunction of the corpora cavernosa [2]. Many studies have focused on postsurgery penile rehabilitation through the application of stem cell therapy, gene therapy, and even small-molecule treatment [1, 3]. However, most of these remain in the experimental stage and require further study. Currently, no causal approaches exist to restore erectile function after radical pelvic surgery [4].

It is well known that corporal fibrosis acts as a major factor in the pathophysiology of ED caused by CN damage [5]. Thus, the antipenile fibrosis properties of HJI have also been studied. Chitaley et al. first discovered that Ras homolog A (RhoA)/Rho-associated protein kinase (ROCK) signaling plays an important role in cavernosal vasoconstriction to inhibit penile tumescence independent of the nitric oxide (NO) pathway [6]. In recent years, RhoA/ROCK in post-prostatectomy ED has become a major focus of investigation [5, 7–11], which can act on smooth muscle to affect erectile function and attenuate cavernous fibrosis.

In China, traditional herbs or formulas to treat ED are widespread and recognized for their effectiveness [12, 13]. The basic pathogenesis of ED after radical prostatectomy, in terms of traditional Chinese medicine, is considered a Qi deficiency and dysregulated blood stasis [14]. The HJI recipe, which consists of nine commonly known herbs, can effectively invigorate the Qi and activate blood circulation. We previously found that treatment with HJI combined with tadalafil was more effective for treating moderate ED than treatment with phosphodiesterase 5 inhibitors (PDE5i) alone [14]. Furthermore, we previously found that HJI effectively alleviated corpus cavernous smooth muscle cell fibrosis and phenotypic changes in rats kept in a hypoxic environment, which is important [15, 16]. However, the pharmacological activity through which HJI improves erectile function is currently unknown. Clinical treatment with HJI has been found to significantly improve erectile function and attenuate cavernosal fibrosis and phenotypic modulation. In this study, we investigated whether HJI could protect the corpus cavernosum from CN injury in a rat model. Expanding our understanding of the drug mechanism will provide a useful strategy for the clinic. Here, we explored the changes in RhoA pathway proteins expression and the degree of fibrosis in the penis tissue of rats with bilateral CN crush injury (BCNI) and the regulatory effects of HJI on ED.

## 2. Materials and Methods

### 2.1. Animals and Grouping

A total of fifty adult male Sprague-Dawley (SD) rats (12-week-old, 350–400 g) with normal erectile function were included in this study and were purchased from the Laboratory Animal Center of Zhejiang Chinese Medical University, China. All animal studies were performed according to the Guide for the Care and Use of Laboratory Animals of the National Institutes of Health. The protocol was approved by the Animal Experimental Ethics Committee of Zhejiang Chinese Medical University. The rats were weighed, randomly divided into five groups, and labeled with picric acid. The rats were divided into the following groups: normal control (NC) + saline; BCNI model (M) + saline; M + low-dose of HJI (HL) (2.835 g/kg/day); M + medium-dose HJI (HM) (5.67 g/kg/day); and M + high-dose HJI (HH) (11.34 g/kg/day). All rats were housed in cages at a temperature of 22 ± 1°C with a 12-h light/dark cycle.

### 2.2. Surgical Procedure

**Figure 1:**
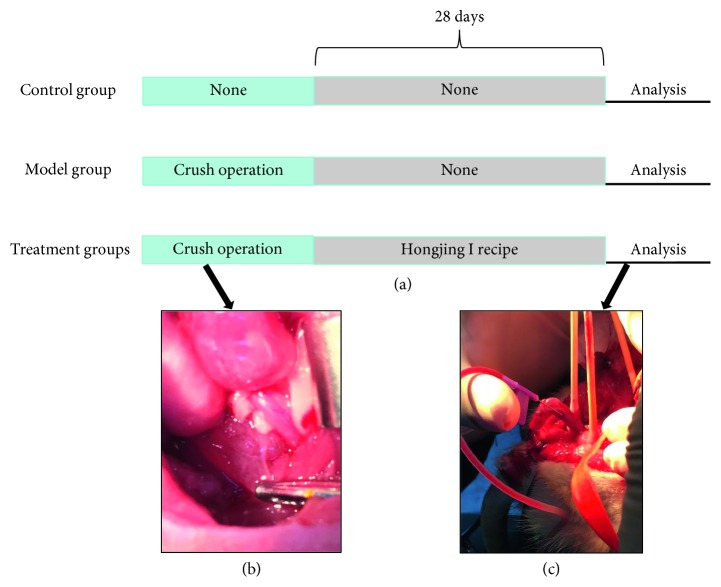
Experimental protocol for model establishment and treatment processes. (a) First, each group of rats was subjected to corresponding modeling treatment, followed by 28 days of drug treatment or saline treatment. (b) Hemostatic forceps clips the CN. (c) Blood reflux can be seen after intubation of the corpus cavernosum.

Rats in the M and HJI treatment groups were subjected to bilateral cavernous nerve crush injury under aseptic conditions. Rats were anesthetized by intraperitoneal injection with 3% sodium pentobarbital solution (50 mg/kg). The procedure was performed as previously described [17]. Before the crush injury, the CN was electrically stimulated while the penis was erect to make sure the correct nerve was isolated. The injury was induced by applying hemostatic forceps to the nerve at ∼3–5 mm distal to the major pelvic ganglion (Figure 1(b)). Forceps were held closed twice for 60 s each to induce a moderate nerve crush injury. There was a washout period of 3 d after the end of the experimental period.

### 2.3. Preparation and Application of Drugs

HJI was composed of *Rhodiola rosea* (Hong-Jing-Tian, batch no. 18061253) 15 g, *Radix astragali* (Zhi-Huang-Qi, batch no. 18081763) 20 g, *Codonopsis pilosula* (Chao-Dang-shen, batch no. 18091823) 15 g, *Angelica sinensis* (Quan-Dang-Gui, batch no. 18111803) 12 g, *Salvia miltiorrhiza* (Dan-shen, batch no. 18100083) 15 g, Raidix Paeoniae Alba (Bai-Shao, batch no. 18120903) 15 g, *Lycium chinense* Miller (Gou-Qi, batch no. 18120903) 15 g, *Epimedium brevicornu* (Yin-Yang-Huo, batch no. 18091753) 10 g, *Cyathula officinalis* (Chuan-Niu-Xi, batch no. 18101783) 12 g, which were listed in Table 1. All granulates were purchased from Jiangyin Tianjiang Pharmaceutical Co., LTD. All the traditional Chinese medicine formula granules of HJI are weighed and added to a clean 2 L beaker. The boiling pure water is used as a solvent to dissolve the granules so that the final concentration of the HJI formula granule solution is set at 0.55 g/ml. In order to get the best dissolution of all the granules, a glass rod is used for stirring for 10 minutes. Each time, 2 liters of the red ginseng formula granule solution was prepared, and then, it was used up and then stored in a refrigerator at 4°C. According to the ratio of rat to human body surface area, the dosage required for each kilogram of rats was converted (the rat/human coefficient was 6.3 as the concentration of HJI low-dose group). Then, according to the coefficients 12.6 and 25.2, the dosage concentrations of medium and high dosage of HJI were calculated, which were 2.835 g/kg, 5.67 g/kg, and 11.34 g/kg, respectively.

### 2.4. ICP/Mean Arterial Pressure Examination

Rats were anesthetized by intraperitoneal injection of 3% sodium pentobarbital (50 mg/kg), and the CN below the crushed segment was stimulated with a silver bipolar electrode. Subsequently, a 25 G needle containing 100 U/ml heparin solution was cannulated into the right shaft of the penis (Figure 1(c)). Polyethylene-50 tubing was used to connect the needle to an MP160 pressure transducer (Biopac Systems, Inc., Goleta, CA, USA). The following stimulus parameters were used: voltage, 5 V; frequency, 20 Hz; and pulse width, 5 ms. The duration of each stimulation was 60 s with a 5–10 min rest period between stimulations, and each rat underwent three stimulations. To record the mean arterial pressure (MAP), the same tubing was cannulated into the unilateral common carotid artery after making a medium incision in the neck. Erectile function was evaluated based on baseline ICP, maximal ICP, the ratio of maximal ICP to MAP (ICP/MAP), and the ratio of the area under the curve to MAP (AUC/MAP). The ICP operator tested the animals in a blinded fashion.

### 2.5. Collection of Penile Tissue

After measuring ICP *in vivo*, the penises were harvested. The middle region of the skin-denuded penile shaft was fixed in 4% paraformaldehyde and then embedded in paraffin for histological studies. The remaining tissue was stored at −80°C for western blot analysis.

### 2.6. Masson's Trichrome Staining and Immunofluorescence

Paraffinized tissue was sliced to a thickness of 5 *μ*m for immunofluorescence staining. For immunohistochemical staining, the slides were incubated overnight with rabbit primary antibodies against RhoA (1 : 200), ROCK2 (1 : 250), and ROCK1 (1 : 200; Abcam, Cambridge, UK). Next, the sections were immersed in a 1 : 500 dilution of Alexa Fluor 488-conjugated secondary antibody (Immunoway, Plano, TX, USA) for 1 h at room temperature. Nuclear staining was performed with 4′, 6-diamidino-2-phenylindole (DAPI; Beyotime, Shanghai, China). To detect the ratio of rat cavernosum smooth muscle to collagen, cavernosum tissue sections were stained with Masson's trichrome (Solarbio, Beijing, China). Smooth muscle cells were stained red, whereas collagen fibrils were stained blue. Histomorphometric analyses of both Masson's trichrome staining and immunofluorescence (IF) staining were performed using ImagePro Plus 6.0.

### 2.7. Western Blot Analysis

Western blotting was performed as described in our previous study [18, 19]. In brief, penis tissues were pulverized using a T10 basic homogenizer (IKA, Staufen, Germany). The tissue fragments were then lysed in cell lysis buffer for at least 0.5 h on ice and centrifuged at 12,000 × g for 15 min at 4°C. Total tissue protein from rat penises was determined using a bicinchoninic acid (BCA) protein assay kit (Beyotime, Jiangsu, China) following the manufacturer's protocol. Next, 10 *μ*g protein was separated by ∼6–10% SDS-PAGE and transferred onto polyvinylidene difluoride (PVDF) membranes (Bio-Rad Laboratories, Hercules, CA, USA). After blocking with Tris buffer solution containing 5% nonfat milk for 1 h at 25–30°C, samples were incubated with the indicated primary antibodies in phosphate-buffered saline supplemented with Tween-20 at 4°C overnight. The primary antibodies used were anti-RhoA (1 : 5000), anti-ROCK1 (1 : 5000), anti-ROCK2 (1 : 1000; Abcam), anti-Collagen I (1 : 1000; Abcam), and anti-*β*-actin (1 : 1000; Immunoway, China). *β*-Actin was used as a loading control. Samples were then incubated with horseradish peroxidase-conjugated secondary antibodies at room temperature for 1 h. Next, the membranes were treated with an enhanced chemiluminescence reagent (Beyotime). Images were captured with the FluorChem R imaging system and analyzed using ImagePro Plus 6.0.

### 2.8. Statistical Analysis

Statistical analysis was performed by GraphPad Prism Version 6 (GraphPad Software, Inc., San Diego, CA, USA). All experimental data were collected in a blinded fashion; the operator and designer were blinded to each other. All data were expressed as the mean ± standard deviation (SD) and analyzed by one-way ANOVA with Tukey's *post hoc* test for multiple-group comparison. A value of *P* < 0.05 was considered statistically significant.

## 3. Results

### 3.1. Effects of HJI on Erectile Function in Rats with BCNI-ED

**Figure 2:**
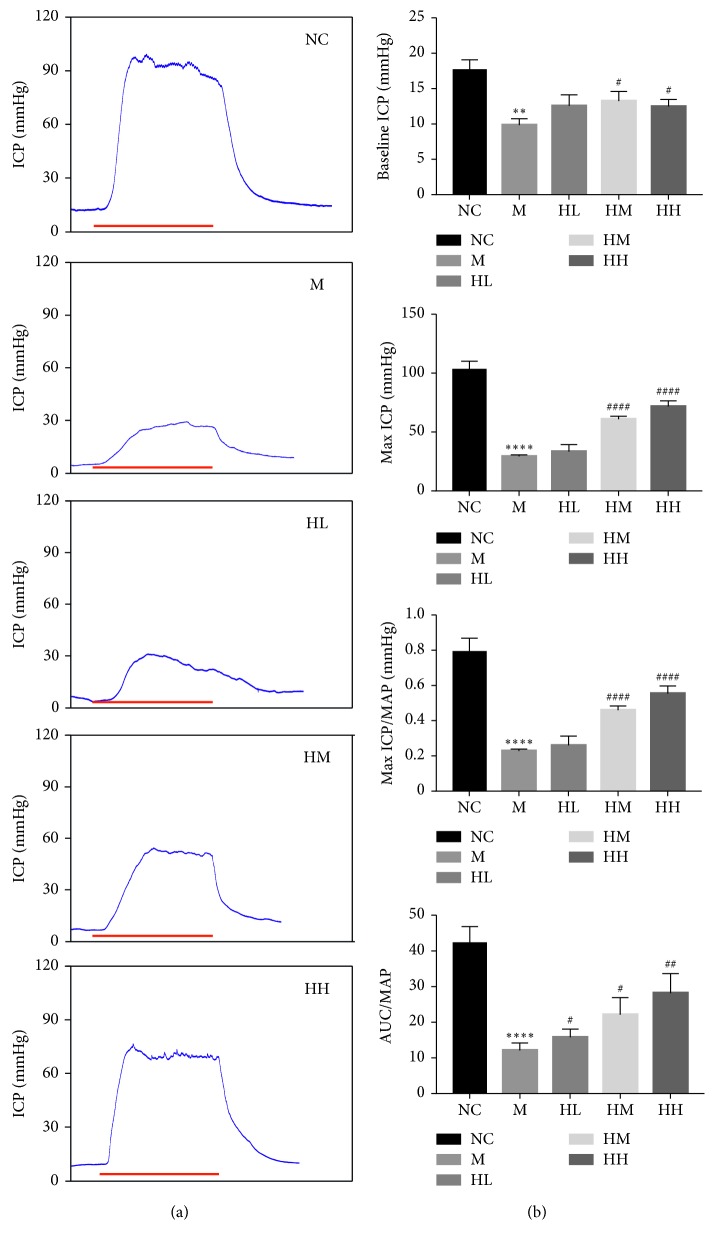
Erectile function of all rats after 4 weeks of treatment was measured by electric stimulation of cavernous nerves. (a) Representative intracavernous pressure (ICP) values in the normal control (NC), bilateral cavernous nerve crush injury model M, low-dose HJI (HL), medium-dose HJI (HM), and high-dose HJI (HH) groups; blue lines means pressure curves, and red lines means 1 min electrical stimulation. (b) Baseline ICP, max ICP, max ICP/mean arterial pressure (MAP) ratio, and area under the curve (AUC)/MAP ratio in response to electric stimulation of the CN, respectively, in all rats. Data are expressed as the mean ± SD. ^*∗∗*^*P* < 0.01 and ^*∗∗∗∗*^*P* < 0.0001 vs NC group; ^#^*P* < 0.05, ^##^*P* < 0.01, and ^####^*P* < 0.01 vs M group.

Erectile function was evaluated by measuring baseline ICP, maximal ICP, maximal ICP/MAP, and the ratio of the area under the ICP curve to MAP at stimulating voltage of 5V (Figure 2). All erectile function variables were significantly lower in the M group than those in the NC group (*P* < 0.01). Compared to that in the M group, the baseline ICP was significantly improved in the HM and HH groups (*P* < 0.05). In addition, the maximal ICP and maximal ICP/MAP in the HM and HH groups were significantly increased compared to those in the M group (*P* < 0.0001). The AUC/MAP was also significantly improved in the HM and HH groups (*P* < 0.05). No statistically significant differences were found between the M and HL groups in terms of baseline ICP, maximal ICP, or maximal ICP/MAP. However, the HL group did show an increase in the AUC/MAP (*P* < 0.05). Furthermore, ICP cannot restore to normal level in the HM and HH groups.

### 3.2. Effects of HJI on Smooth Muscle and Collagen Levels in the Midshaft Penile Tissues of Rats with BCNI-ED

**Figure 3:**
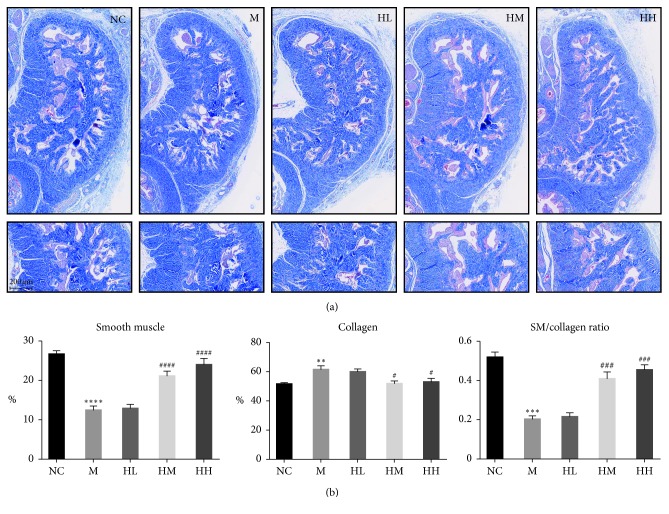
Masson's trichrome staining of midshaft penile tissues (100x magnifications). (a) Representative Masson's trichrome staining of the normal control (NC), bilateral cavernous nerve crush injury model M, low-dose HJI (HL), medium-dose HJI (HM), and high-dose HJI (HH) groups (scale bar = 100 *μ*m). (b) Effect of HJI treatment on the proportion of smooth muscle, the proportion of collagen, and the ratio of SM to collagen in the corpus cavernosum. ^*∗∗*^*P* < 0.01, ^*∗∗∗*^*P* < 0.001, and ^*∗∗∗∗*^*P* < 0.0001 vs NC group; ^#^*P* < 0.05, ^###^*P* < 0.001, and ^####^*P* < 0.0001 vs M group.

Cavernosal fibrosis is an important pathological process leading to ED [20] and can be determined by Masson's trichrome staining. Compared to the NC group, the M group exhibited a clear decrease in corporal smooth muscle content and an increase in collagen deposition (*P* < 0.0001) (Figure 3(a)). Smooth muscle content was significantly increased, and collagen area was significantly decreased, in the HM and HH groups (*P* < 0.01) (Figure 3(b)). In addition, the smooth muscle/collagen ratio was significantly decreased in the M group compared to that in the NC group, and the HM and HH treatment groups showed a significantly improved smooth muscle/collagen ratio (*P* < 0.001) (Figure 3(b)). Furthermore, there was no statistical significance in the smooth muscle, collagen, and SM/collagen content of the HH group and NC group.

### 3.3. Effects of HJI on the Expression of RhoA in BCNI Rats

**Figure 4:**
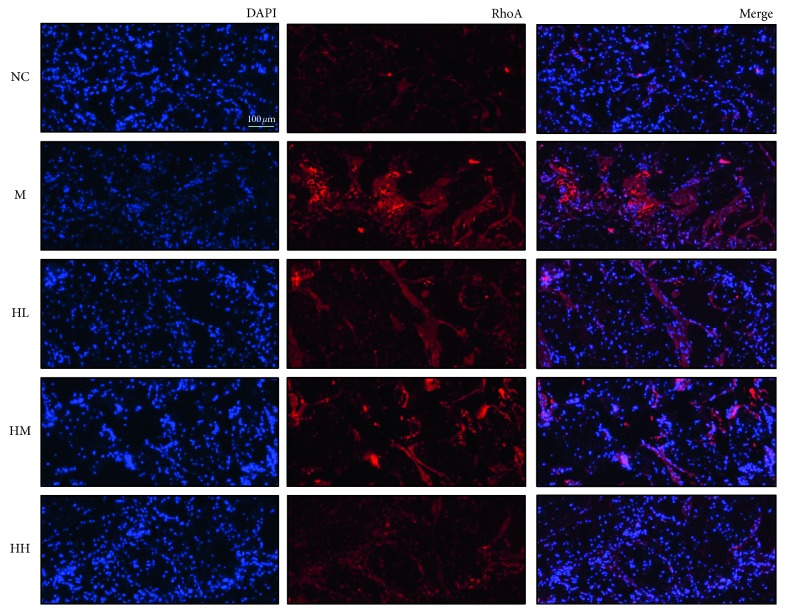
Effects of HJI on the expression of RhoA in BCNI rats through immunofluorescence staining (200x magnifications). Immunofluorescent staining shows the colocalization of RhoA (red) and neuronal cells (blue) in the corpora cavernosa. DAPI: 4′,6-diamidino-2-phenylindole; NC:  normal control; M: bilateral cavernous nerve crush injury model; HL: low-dose HJI; HM: medium-dose HJI; HH: high-dose HJI.

The expression of RhoA in the model group was significantly increased after CN injury as shown by IF staining, and RhoA expression decreased after treatment in a dose-dependent manner. Accordingly, RhoA expression in the HH group was the lowest (Figure 4).

### 3.4. Effects of HJI on the Expression of ROCK1 in BCNI Rats

**Figure 5:**
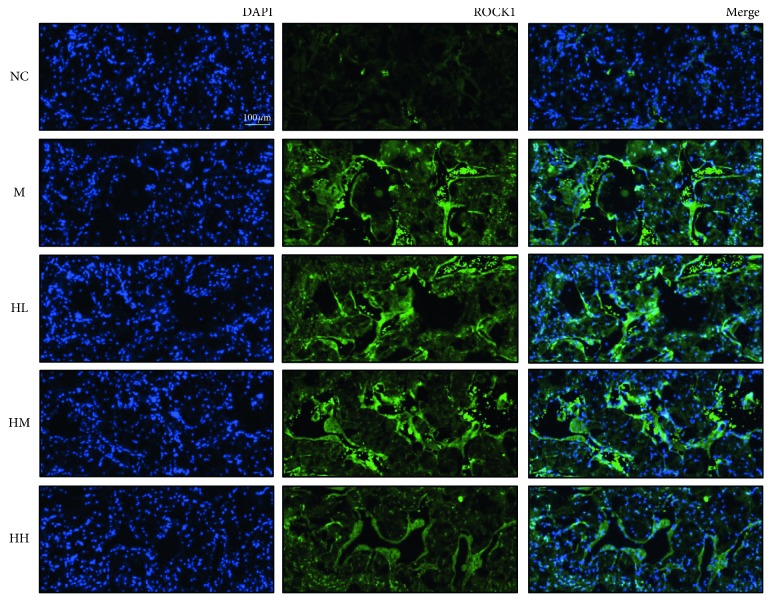
Effects of HJI on the expression of Rho-associated protein kinase 1 (ROCK1) in BCNI rats through immunofluorescence staining (200x magnification). Immunofluorescence staining shows the colocalization of ROCK1 (green) and neuronal cells (blue) in the corpora cavernosa. DAPI: 4′,6-diamidino-2-phenylindole; NC: normal control; M: bilateral cavernous nerve crush injury model; HL: low-dose HJI; HM: medium-dose HJI; HH: high-dose HJI.

The expression of ROCK1 was significantly increased in the model group after BCNI. However, its expression was not majorly affected by increased drug administration (Figure 5).

### 3.5. Effects of HJI on the Expression of ROCK2 in BCNI Rats

**Figure 6:**
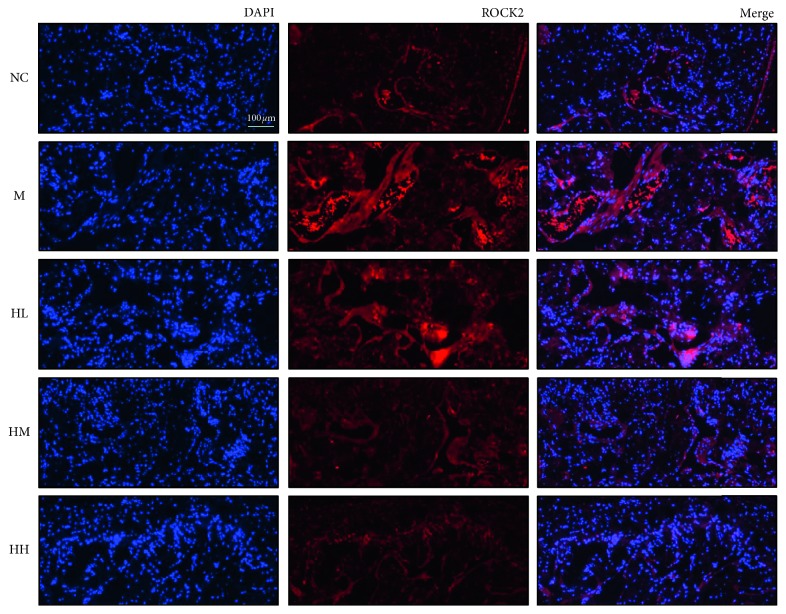
Effects of HJI on the expression of Rho-associated protein kinase 2 (ROCK2) in BCNI rats through immunofluorescence staining. Immunofluorescent staining shows the colocalization of ROCK2 (red) and neuronal cells (blue) in the corpora cavernosa (200x magnification). DAPI: 4′,6-diamidino-2-phenylindole; NC: normal control; M: bilateral cavernous nerve crush injury model; HL: low-dose HJI; HM: medium-dose HJI; HH: high-dose HJI.

The expression of ROCK2 was significantly increased in the model group after BCNI. Notably, the expression of ROCK2 decreased, similar to our observations for RhoA, upon treatment with increasing drug concentrations; the expression of ROCK2 showed the greatest decrease in the HH group (Figure 6).

### 3.6. Effects of HJI on the Expression of RhoA, ROCK1, and ROCK2 in the Penis Tissues of BCNI-ED Rats

**Figure 7:**
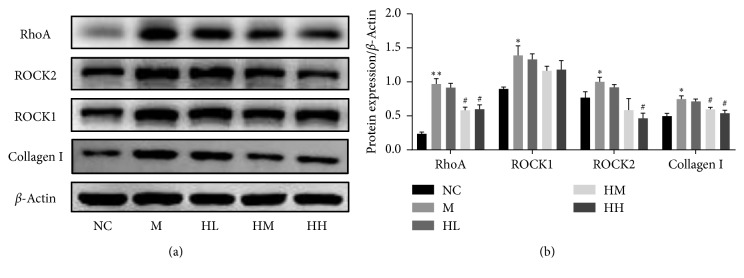
Effect of HJI on RhoA, ROCK1, ROCK2, and collagen I activity in rat corpus cavernosum tissues. (a) Western blot analysis showing the protein expression levels of RhoA, ROCK1, ROCK2, and collagen I in rat corpus cavernosum tissues. (b) Data are presented as the relative density of RhoA, ROCK1, ROCK2, and collagen I expression with *β*-actin as the loading control. Values are presented as the mean ± standard deviation. ^*∗*^*P* < 0.05 and ^*∗∗*^*P* < 0.01 vs NC group; ^#^*P* < 0.05 vs M group. NC: normal control; M: bilateral cavernous nerve crush injury model; HL: low-dose HJI; HM: medium-dose HJI; HH: high-dose HJI.

RhoA, ROCK1, ROCK2, and collagen I expression were quantified by western blotting (Figure 7). Compared to that in the NC group, the relative expression of above proteins was increased in the M group (*P* < 0.05). In the HL treatment group, there were no significant differences in RhoA, ROCK1, ROCK2, and collagen protein expression. However, RhoA, ROCK2, and collagen I protein expression was markedly decreased in the HM and HH groups (*P* < 0.05). ROCK2 protein expression was significantly higher in the HH group only. No significant differences in RhoA, ROCK1, ROCK2, or collagen I expression were found in the HL treatment group. Compared with the control group, RhoA could not return to normal level in the treatment group. Furthermore, there was no significant difference in collagen I expression compared the NC group with the HH group.

## 4. Discussion

Apoptosis, collagen deposition, and the fibrosis of smooth muscle and endothelial cells in the corpus cavernosum may occur during neurapraxia mediated by CN injury. The reduced expression of nitric oxide synthase (NOS) and changes in the signal intensity of transforming growth factor-*β*1 (TGF-*β*1) and RhoA are closely related to the above pathological process [5, 9, 10, 21, 22]. Clinical studies suggest that the erection induced by sexual stimulation and nonsexual stimulation (spontaneous erection at night) in patients with radical prostatectomy is significantly reduced [23], resulting in a relatively ischemic/hypoxic state of the corpus cavernosum during convalescence, which is an important factor leading to organic changes such as penile fibrosis. The corpus cavernosum is the final effector organ of the erectile response. The corpus cavernosum smooth muscle cells are the basis of the corpus cavernosum; they also control the relaxation and contraction of the corpus cavernosum. When tissue structures are destroyed due to fibrosis, erectile function cannot be restored, even after the improvement of neurological symptoms. Therefore, the prevention and treatment of cavernous fibrosis is of great significance to regain normal erectile function upon neurapraxia due to CNI. A major purpose of this study was to analyze the effect of HJI on fibrosis in the cavernous penile tissue of rats. We found that BCNI caused significant cavernous corpus remodeling, as shown by the observed increase in cavernous fibrosis and collagen deposition in the model and HL treatment groups compared to those in the sham, HM treatment, and HH treatment groups. Medium-dose treatment and high-dose treatment were found to increase smooth muscle content while reducing the deposition of the fibrosis-related protein collagen I compared to those in the model group, indicating that HJI could reduce corpus cavernosum fibrosis and improve erectile function.

The RhoA/ROCK pathway was discovered in the 1990s and has since been proven to play an important role in regulating erectile function [6]. The ROCK signaling pathway maintains the contractile state of corpus cavernosum smooth muscle, and phosphorylated ROCK allows myosin light chain to stay phosphorylated and thus actin-contracted [24]. Early studies on ED did not differentiate between the two ROCK1 and ROCK2 subtypes, but increasingly exhaustive research has shown that they play distinctive roles in ED caused by different etiologies [7]. An early view was that the upregulation of ROCK2 played a major role in CNI-ED, but subsequent studies have confirmed that the high expression of ROCK1 is also closely related to cavernous fibrosis after CNI-ED [5, 9, 10, 21]. Studies by Cho et al. [5], Song et al. [11], and Cho et al. [21] found that ROCK1 expression was increased in SD rats after the CN was crushed and transected. However, Gratzke et al. [10] found that ROCK1 was not upregulated after CNI, and whether the degree of damage caused by different CNI methods affects the expression of ROCK1 remains to be studied. Therefore, we included both ROCK1 and ROCK2 in the present study. Unlike the above studies, we found that both isoforms showed an increase after 28 days of CN injury. In addition, no clear correlation has been shown for the effect of age on ED in rats compared to that in humans. It is also uncertain whether changes in the expression of ROCK1 and ROCK2 at different time points represent the changes of ROCK subtypes in the acute, subacute, and chronic stages after surgery.

Cavernous injury, diabetes, and other types of ED-associated disorders are not significant for first-line clinical treatment with PDE5i, so more treatment regimens and means are needed for Ed treatment [25]. At present, the relationship between RhoA/ROCK signaling and ED has attracted the attention of many researchers. RhoA/ROCK pathway activation is closely related to erectile function, as well as cavernosal and penile fibrosis [20, 26]. The inhibition of ROCK1 or its downstream molecules, LIMK2 and Coflin, has been shown to effectively attenuate penile fibrosis. The current results showed that ROCK1 expression was decreased in the drug group, but not significantly; whether HJI affects the downstream molecules of ROCK1 requires further study. ROCK2 plays an important role in fibrosis in organs such as the heart, kidney, and lung [27–29]. The degree of fibrosis is known to be reduced through ROCK2 gene knockout or inhibition [9, 30]. Moreover, ROCK2 expression is upregulated in ED; erectile function can be markedly improved by knocking down the gene or treatment with ROCK inhibitors. Fluorescence and western blotting results showed that the activated ROCK2 signaling pathway was significantly inhibited after drug treatment. The relationship between ROCK2 and fibrosis in ED caused by CN injury has not been researched often. Future studies should investigate whether ROCK2 elevation can regulate cavernous fibrosis. Based on the small sample population for ED pathology [31], expression of ROCK2 was significantly upregulated in tissue from men with ED. Drug can inhibit ROCK2 efficiently, which may have important benefits for the treatment of ED. In this study, activated RhoA/ROCK2 signaling in CNI rats was significantly inhibited by drug treatment. Therefore, we speculate that the antifibrosis effect of this drug could have been through the action of ROCK2 or other pathways. Because RhoA signaling also plays an important role in regulating erectile function independent of the classical NO signaling pathway, several key molecules in the RhoA pathway, namely, RhoA, ROCK1, and ROCK2, were also analyzed in this study. The results showed that RhoA, ROCK1, and ROCK2 were highly expressed in the spongy tissue of BCNI rats, consistent with the results of previous studies. RhoA and ROCK2 in rat cavernosum tissues showed decreased expression after intervention with HJI, but the expression of ROCK1 was not significantly affected by our treatment. As the most commonly used index for evaluating erectile function [32], the ICP indicated that HM and HH treatments did not fully improve erectile function to normal levels. HJI plays a role in the partial pathogenesis of BCNI-induced ED; however, HJI may achieve better therapeutic effects if it is combined with PDE5i drugs or other clinical treatments. This study showed that the improvement of CNI-ED by HJI treatment may be related to the inhibition of RhoA/ROCK2 signaling activation and amelioration penis fibrosis, and that might be the mechanisms through which HJI protects erectile function.

## 5. Conclusions

Our results demonstrated that the Chinese medicine HJI ameliorated erectile function and inhibited penis fibrosis in BCNI rats. Furthermore, the effects of HJI on erectile function may be caused, at least in part, by the suppression of RhoA/ROCK2 signaling and cavernosum fibrosis. However, the precise mechanism of action of this medicine must be clarified through further investigations.

## Figures and Tables

**Table 1:** Herbal ingredients of Hongjing I recipe.

Chinese name	Full scientific name	Part used	Proportion (%)
Hong-Jing-Tian (紅景天)	*Rhodiola rosea*	Dried root	4.8
Zhi-Huang-Qi (炙黃芪)	*Radix astragali*	Dried root	19.1
Chao-Dang-shen (炒黨參)	*Codonopsis pilosula*	Dried root	14.3
Quan-Dang-Gui (全當歸)	*Angelica sinensis*	Dried root	15.3
Dan-shen (丹參)	*Salvia miltiorrhiza*	Dried root	9.6
Bai-Shao (白芍)	*Raidix paeoniae Alba*	Dried root	4.8
Gou-Qi (枸杞)	*Lycium chinense* Miller	Dried fruit	19.1
Yin-Yang-Huo (淫羊藿)	*Epimedium brevicornu*	Dried leaf	1.6
Chuan-Niu-Xi (川牛膝)	*Cyathula officinalis*	Dried root	11.5

## Data Availability

The data used and analyzed in the present article are available from the corresponding author on reasonable request.
